# Pluripotency genes of mammals: a network at work

**DOI:** 10.3389/fbioe.2025.1578499

**Published:** 2025-06-12

**Authors:** Ranieri Cancedda, Maddalena Mastrogiacomo

**Affiliations:** ^1^ Dipartimento di Medicina Sperimentale, Università degli Studi di Genova, Genova, Italy; ^2^ Dipartimento di Medicina Interna e Specialità Mediche (DIMI), Università degli Studi di Genova, Genova, Italy

**Keywords:** stem cells, pluripotent cells, pluripotency genes, Oct4, Sox2, Nanog

## Abstract

Pluripotency, i.e., the ability to differentiate into cells of all three germ layers, is a transient state of early embryonic cells. In mammals, during progression from pre-implantation to post-implantation stage, pluripotent cells undergo different state transitions characterized by changes in gene expression and development potential. These developmental states include: (i) a naive pluripotency (pre-implantation embryonic stem cells, or ESCs), (ii) an intermediate condition (formative state), and (iii) a primed pluripotency (late post-implantation ESCs derived from epiblasts also named EpiSCs). The transitions are regulated by an interconnected network of pluripotency-related genes. Transcription of genes such as *Oct4, Sox2*, and *Nanog* is crucial for obtaining and maintaining pluripotency. These three factors form an autoregulatory loop by binding to each other’s promoters to activate their transcription. Other factors play a significant ancillary role in the transcription factor network preserving cell pluripotency. In the review, we will also mention some of the more relevant cytokines, molecules, signaling pathways, and epigenetic modifications that induce and control pluripotency gene expression. The main goal of this review is to bridge the gap between the fields of genetics and stem cell biology and to set the ground for the application of this knowledge to the development of strategies and drugs to be used in a clinical environment.

## Early embryogenesis progression

The mammalian female oocyte is one of the largest cells in the body. Its cytoplasmic components, mRNAs, microRNAs, and proteins are unevenly distributed in the cell and one can distinguish an animal and a vegetal pole, ultimately defining future embryo axes. After fertilization, the oocyte completes the meiosis. This is followed by the totipotent zygote formation and few rounds of cell division. At the time of these initial cell cleavages, before the onset of protein synthesis, and the increase of the embryo volume, different portions of the maternal cytoplasm are associated with nuclei, in principle identical, thus giving origin to different cells. After completing three cleavage rounds, when the embryo is at the 8-cells (named blastomeres) stage, cells undergo an increase in cellular adhesion known as compaction and the embryo becomes a morula. When protein synthesis begins, new proteins are synthesized by the maternal mRNAs whereas maternal microRNAs and maternal and newly synthesized proteins promote epigenetic modifications, mainly methylation and histone reorganization in the cell DNA and chromatin. The epigenetic modifications can lead to the silencing of some genes coding for transcription factors acting in the initial phase of embryogenesis and to the activation of initially silent genes coding for a different set of transcription factors. Other proteins typically synthesized at this stage are soluble modulators such as growth factors and cytokines and their receptors. The continuous autocrine and paracrine crosstalk between the different embryo cells occurring at this stage determine its progression leading to the formation of the fluid-filled blastocyst cavity and the blastocyst inner cell mass. Eventually a gastrula is formed, the embryonic anterior-posterior and dorso-ventral axes become established, and the three germ layers start to be specified.

Coinciding with the embryogenesis progression, molecular and spatial-temporal mechanisms employed by totipotent blastomeres, capable to give rise to all embryo tissues as well as all extra-embryonic lineages, such as the placenta and the yolk sac, acquire more restricted signatures specific for cell lineages. The first of these lineage decisions is the formation of the inner cell mass (ICM) that gives rise to cells of all embryo tissues but not to cells of extra-embryonal tissues, whereas other blastocyst cells contribute exclusively to extra-embryonal tissues. Gastrula cells have a more restricted genetic signature and give rise only to cells of tissues derived from a single germ layer: ectoderm, mesoderm, and endoderm.

**FIGURE 1:**
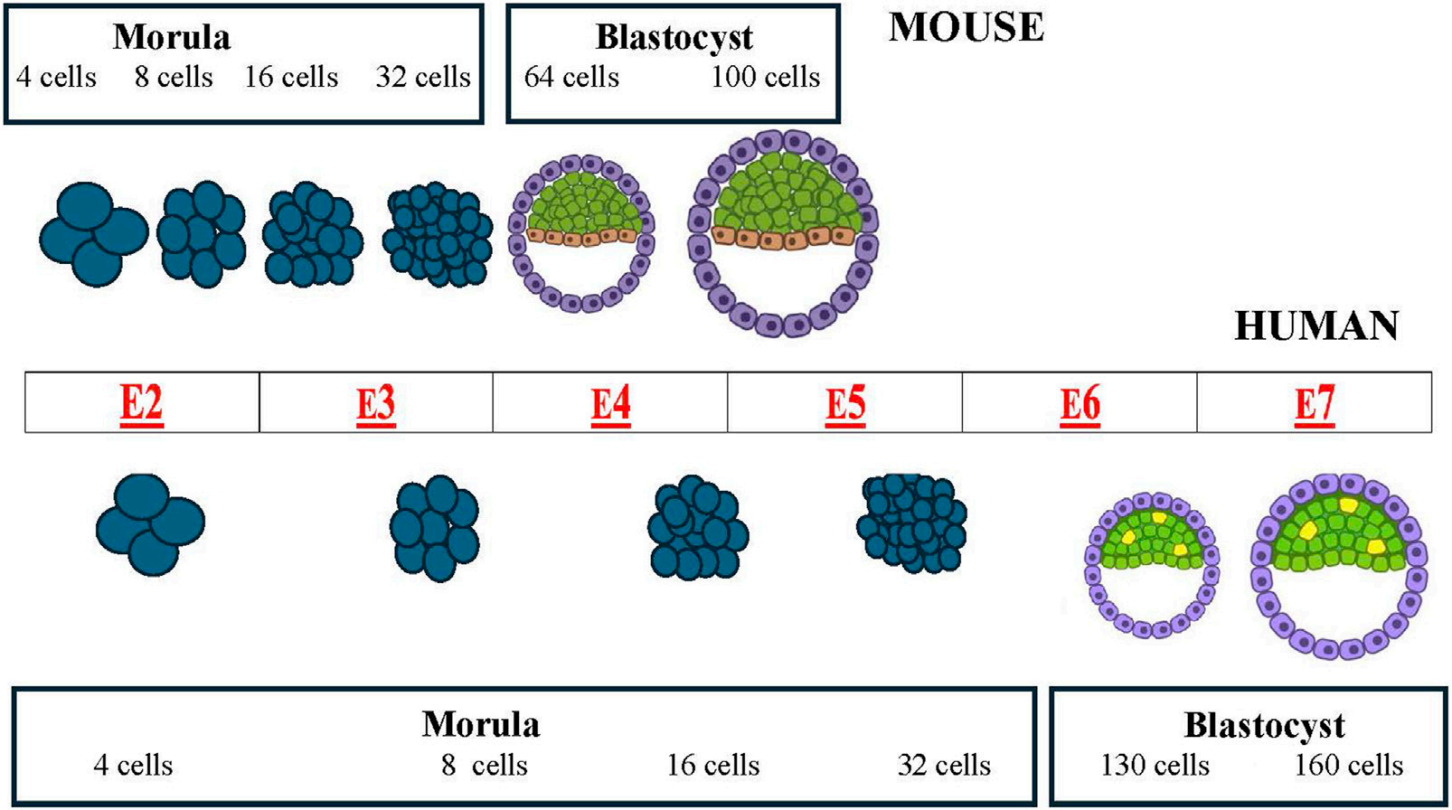
Comparative schematic of mouse (top) and human (bottom) pre-implantation development (from morula to blastocyst, showing the timing of the different morphological stages. The center table (red letters) shows the embryonic day (E) of the associated event.

**FIGURE 2:**
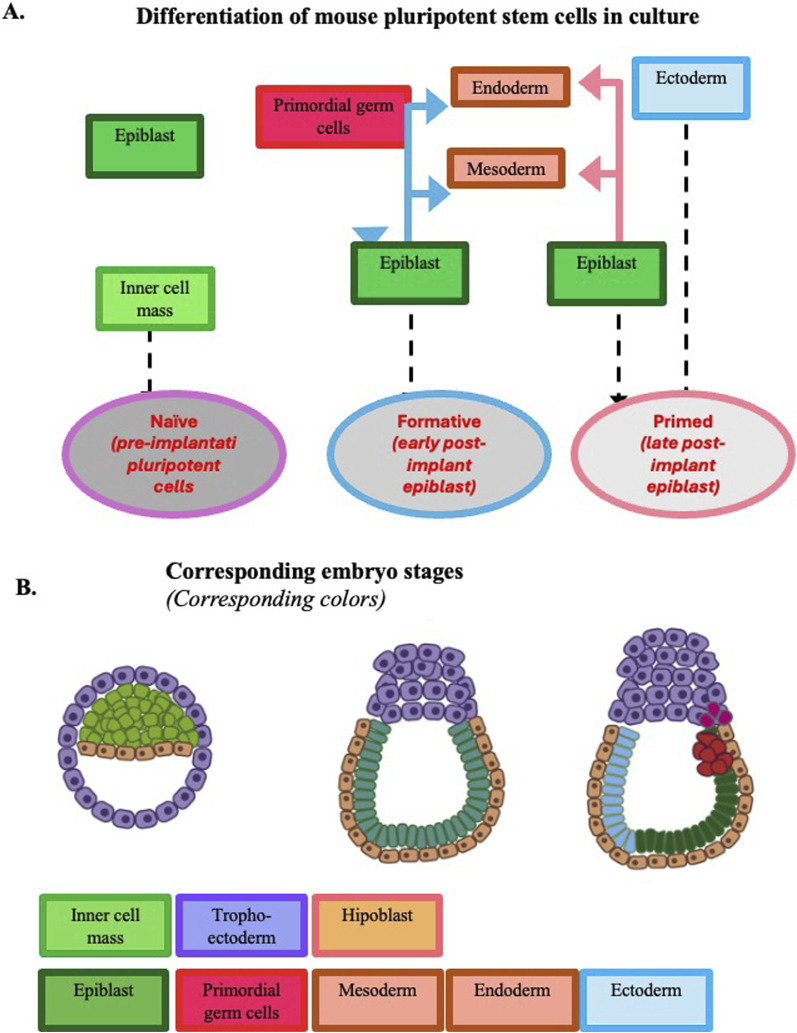
Development potential of mouse pluripotent stem cells expanded in culture. Panel **(A)** Developmental potential at the three pluripotency stages observed *in vitro*. Panel **(B)** Developmental potential of epiblasts during mouse early embryogenesis up to gastrulation. To facilitate the comparison of the pluripotency expressed by the cells *in vitro* and *in vivo*, the same color is adopted in the two panels to identify the different tissues. [Modified from (Pera and Rossant, 2021)].

**FIGURE 3:**
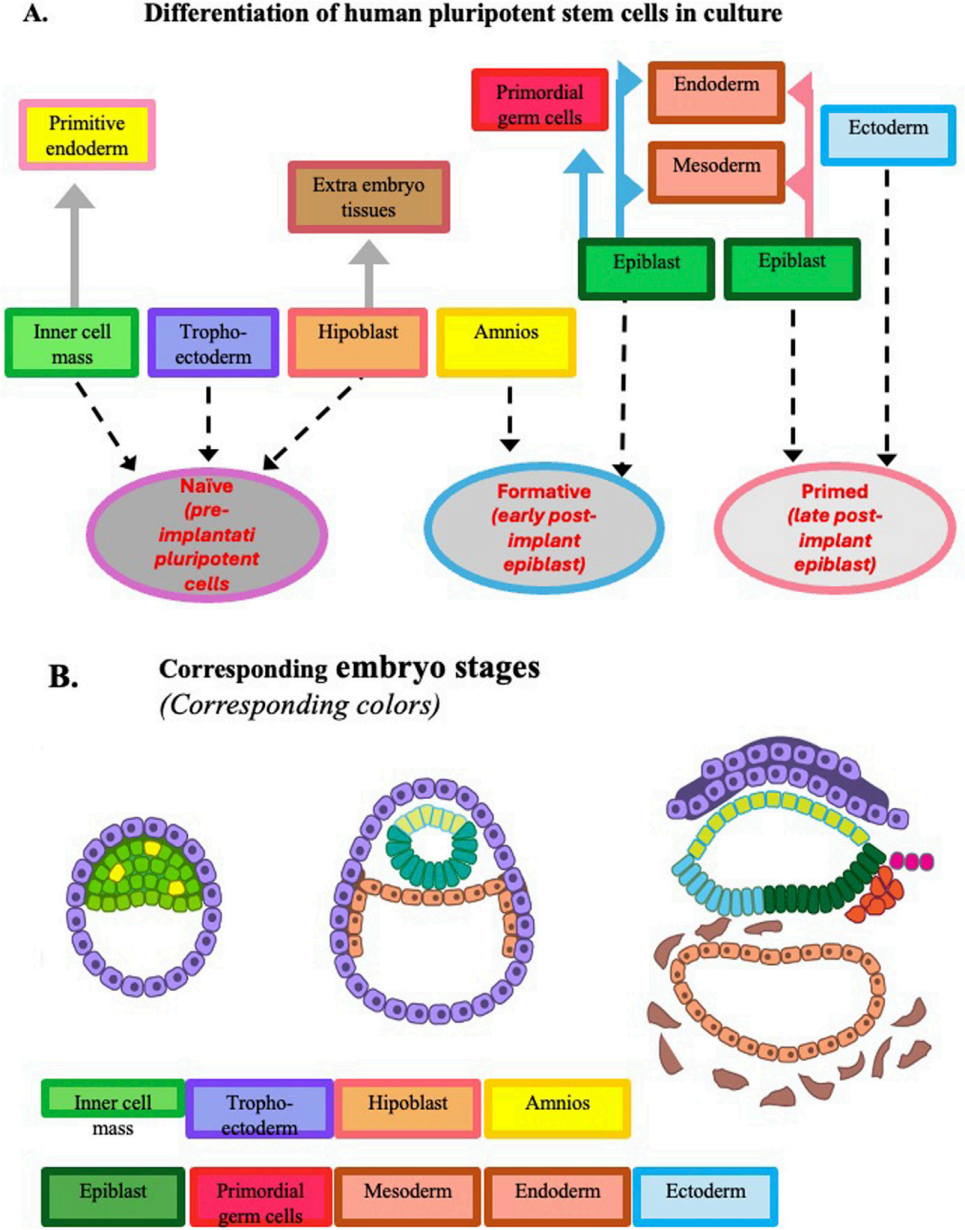
Development potential of human pluripotent stem cells expanded in culture. Panel **(A)** Developmental potential at the three pluripotency stages observed *in vitro*. At variance with the corresponding mouse cells, human naïve pluripotent stem cells can give origin to primitive endoderm and trophoectoderm, and when they reach the formative stage, they can give origin to amniotic cells. Panel **(B)** Developmental potential of epiblasts during human early embryogenesis up to gastrulation. To facilitate the comparison of the pluripotency expressed by the cells *in vitro* and *in vivo*, the same color is adopted in the two panels to identify the different tissues. [Modified from (Pera and Rossant, 2021)].

These basic mechanisms are common to all mammalian embryos. However, with the embryogenesis progression some species differences become evident. When embryogenesis is compared in mice and humans, major differences can be seen regarding the temporal length of the pre-implantation phase (Figure 1) and the spatial organization of the gastrula (Figures 2, 3).

The purpose of this review is to shed some light on the main genetic mechanisms controlling the initial phase of mouse and human embryogenesis not only to expand our knowledge but also to set the ground for the application of this knowledge to a better understanding of how the pathways described relate to human pathology, particularly in oncology (i.e., cancer stem cells, tumor plasticity, and metastatic capabilities). Eventually this should lead to the development of strategies and drugs to be used in a clinical environment.

## State transition of pluripotent cells during early embryogenesis

Embryonic stem cells (ESCs) are usually defined as cells derived from the inner cell mass of a blastocyst, an early embryo, that can develop into any type of cell in the body (pluripotent). This not always true. In the mammalian embryos, during progression from pre-implantation to post-implantation developmental stages, pluripotent ESCs undergo state transitions characterized by changes in gene expression and development potential. These stages include: (i) a naive pluripotency (pre-implantation embryonic stem cells, or ESCs), (ii) an intermediate condition (formative state), and (iii) a primed pluripotency (late post-implantation ESCs derived from epiblasts also named EpiSCs) (Nichols and Smith, 2009; Smith, 2017; Pera and Rossant, 2021). The naive state is characterized by more undifferentiated cells with the potential to differentiate into a wide range of cell types. On the contrary, the primed state is more restricted in terms of differentiation potential. Some recent studies shed light on the molecular mechanisms underlying pluripotency in different mammalian species, including mouse, bovine, and pig, and revealed the existence of conserved genes that play a crucial role in distinguishing between naïve and primed pluripotency states in embryos.

In the proper culture conditions, ESCs derived from the inner cell mass can proliferate *ad infinitum*, retaining pluripotency. The naive mouse ESCs can join and integrate in the morula of a different embryo to contribute to the soma and the germline formation in chimeras. During the early embryo development, pluripotent cells become restricted to the epiblast layer (EpiSCs) before the formation of the three germ layers. After the embryo implant in the uterus, the epiblast cells undergo morphological transitions and changes in gene expression leading up to the onset of gastrulation and lineage differentiation. A more detailed description of mechanisms making and shaping germ layers during gastrulation can be found in (Solnica-Krezel and Sepich, 2012). Pluripotency, i.e., the capacity to differentiate into multiple lineages, is lost by the epiblast after gastrulation when they undergo silencing of core transcription factor genes, activation of differentiation genes, and progressive differentiation into the three germ layers and their derivatives. Up to the gastrulation stage, pluripotent EpiSCs can be obtained and expanded in culture as a self-renewing cell population. EpiSCs are more primed to differentiate (less naïve), and have different growth properties and transcriptional signatures than ESCs [(Hanna et al., 2010) for a review]. When EpiSCs are transplanted in different epiblast locations they change their future fate according to their new position and microenvironment (Beddington, 1982; Tam and Zhou, 1996).

In human, *in vivo* studies like the ones performed with mice are impossible or difficult to execute because of ethical scruples and the technical difficulty to recover embryos immediately post-implantation. Properties of post-implantation human epiblasts were often extrapolated from the pre-implantation state cells based on gene expression and epigenetic modifications. At variance with the mouse blastocyst inner cell mass-derived ESCs which are in the naïve state, the human ESCs are considered to be in a primed phase (Brons et al., 2007; Tesar et al., 2007) similarly to both mouse and human EpiSCs and human iPSCs. These similarities are defined based on several aspects. Mouse ESCs depend on Leukemia Inhibitory Factor (LIF) and Bone Morphogenetic Protein (BMP), whereas human ESCs depend on activin A/nodal (members of a Transforming Growth Factor (TGF) beta sub-family) and Fibroblast Growth Factor 2 (FGF2) signaling pathways (Brons et al., 2007; Li and Belmonte, 2017). Naive mouse ESCs or iPSCs, expanded in the presence of LIF, become comparable to the epiblast regarding gene expression profiles, epigenetic marks, metabolism, and differentiation capacity. Recent findings suggest that in mouse ESCs the turnover of the histone chaperone FACT on chromatin is crucial for regulating the enhancers of formative-specific genes, thereby mediating the naive-to-formative transition (Zhao et al., 2024). However, pluripotency is well defined functionally but is ambiguously defined at the molecular level. Boroviak and colleagues used a multi-species approach to differentiate between fundamental features of pluripotency in mammals and those that exhibit evolutionary plasticity (Boroviak et al., 2015).

Massafret et al. derived human ESCs from single blastomeres of 8-cell embryos and compared their pluripotency state with ESCs derived from whole human blastocysts. No significant differences were observed between blastomere-derived hESCs and blastocyst-derived hESCs for most naïve pluripotency indicators and their trilineage differentiation potential. Nevertheless, blastomer ESCs showed an increased single-cell clonogenicity and a higher expression of naïve pluripotency markers at early passages than blastocyst ESCs. Furthermore, blastocyst ESCs overexpressed a set of genes related to the post-implantation epiblasts. Altogether, these results suggest that blastomer-derived human ESCs, are slightly closer to the naïve end of the pluripotency continuum than blastocyst ESCs (Massafret et al., 2024). However, the differences existing in the gastrulation process in mouse and human peri- and post-implantation embryos are the reflection of differences in gene expression in the naïve to primed pluripotent ESC transition.

In naive-cell-specific culture conditions, human ESCs can be captured in a naive state where they resemble the preimplantation epiblast or in a primed state in which they resemble the post implantation epiblast. This allows the study of preimplantation development *ex vivo* but reportedly leads to chromosomal abnormalities, compromising their utility in research and potential therapeutic applications. The inhibition of the mitogen-activated kinase MEK is essential for the naive state. However, Di Stefano et al. showed that MEK inhibition facilitated the establishment and maintenance of naive human ESCs retaining naive-cell-specific features, such as global DNA hypomethylation, Human Endogenous RetroVirus - beta (HERV-K) expression, and two active X chromosomes (Di Stefano et al., 2018).

Cultured mouse and human ESCs and iPSCs undergo comparable stage transitions during the *in vitro* differentiation to cells of different lineages and can be used as models to investigate gene expression at the different pluripotency cell stages (Figures 2, 3). The naïve to the primed stage occurs *in vitro* after a progression to an intermediate/formative state. Formative pluripotent stem cells having properties between naive and primed pluripotency are defined by a specific transcriptome, a rapid response to lineage induction (including the germline), and an absence of lineage markers. The existence of this intermediate state was initially suggested by investigations on culture conditions promoting the acquisition of germline competence by mouse ESCs. Hayashi et al. demonstrated the generation of primordial germ cell-like cells with robust capacity for spermatogenesis. These cells were generated from ESCs and iPSCs through epiblast-like cells, a cellular state highly similar to pre-gastrulating epiblasts but distinct from EpiSCs (Hayashi et al., 2011). At variance with ESCs, EpiSCs barely contribute to chimeras. Tsukiyama and Ohinata showed that a modified EpiSC culture condition containing the a specific glycogen synthase kinase-3 (GSK3) inhibitor supported a germline-competent pluripotent state intermediate between ESCs and EpiSCs (Tsukiyama and Ohinata, 2014).

Some mechanisms controlling the initial development from naïve to intermediate ESC appear to be common to different species. Interestingly by modulating FGF, TGF-beta, and Wnt pathways, Yu et al. derived pluripotent stem cells from mice, horses, and humans that are permissive for direct *in vitro* induction of cells capable of contributing to intra- or inter-species chimeras *in vivo*. These cells are in a pluripotency state between naive and primed pluripotency and harbor molecular, cellular, and phenotypic features characteristic of formative pluripotency (Yu et al., 2021). However, species differences exist. As example, in human and monkey ESCs the expression of *Nanog* and *Prdm14* (a crucial regulator of mouse primordial germ cells epigenetic reprogramming and pluripotency) persists throughout the transition, unlike in the mouse (Nakamura et al., 2016).

Naive pluripotent ESCs express all the components of the basal pluripotency transcription factor network, including factors such as Oct4, Sox2, Nanog, Klf4, and Esrrb (estrogen-related receptor beta). More details on the nature of these factors and genes and other pluripotency genes will be given in the following paragraphs. At variance with naive cells, EpiSCs express at the same time both pluripotency and lineage-specific markers. EpiSCs can form tissues derived from all three embryonic germ layers but are unable to give rise to germ cells or to contribute to pre-implantation chimera formation. As development of the post-implantation epiblast progresses, the expression of genes associated with neuron differentiation and development, such as *Sox11* begins (Nakamura et al., 2016). Then, as the epiblast prepares for gastrulation, the gene expression profile changes more dramatically. Cells on the verge of gastrulation can be identified based on the expression of genes which are specific lineage markers, including *T (also named Brachyury*), *Gata4*, *Gata6*, and *Mlxl1* (Deglincerti et al., 2016; Nakamura et al., 2016; Shahbazi et al., 2017; Ma et al., 2019). The morphological hallmark of the beginning of gastrulation is the ingress of Oct4/T double-positive cells between the epiblast and the hypoblast at the posterior end of the embryo.

## Genetics of pluripotency induction (iPSC formation)

Pluripotency, i.e., the ability to differentiate into cells of all three germ layers, is a transient state of cells in early embryos. The pluripotency state is regulated by an interconnected network of pluripotency-related genes. In the first decade of this millennium, reprogramming of mouse and human somatic cells to pluripotent cells was obtained by expressing combinations of these pluripotency genes. The 2012 Nobel Prize in Physiology or Medicine was jointly awarded to John B. Gurdon and Shinya Yamanaka for showing that mature cells can be reprogrammed to become pluripotent. In particular, Shinya Yamanaka proved that the introduction of a small set of genes coding for 4 transcription factors, namely, *Oct4*, *Sox2*, *Klf4*, and *c-Myc,* into a differentiated cell was sufficient to revert the cell to a pluripotent state (induced pluripotent stem cells; iPS cells) (Takahashi and Yamanaka, 2006; Takahashi et al., 2007). Almost at the same time, the research team of James Thompson obtained human iPS cells by transfection of a different combination of pluripotency genes, *Oct4, Sox2, Nanog*, and *Lin28*, sufficient to reprogram human somatic cells and to induce iPS cell formation (Yu et al., 2007). The same research group showed that human iPS cells could be used to model the specific pathology seen in a genetically inherited disease (Ebert et al., 2009).

Initial methods to obtain iPS cells employed viral vectors. However, both the viral vector and the transgene sequences after integration into the cell genome could potentially produce insertional mutations interfering with the cell functions. To bypass the potential problem, human iPS cells were obtained with the use of non-integrating episomal vectors (Yu et al., 2009). With non-viral vectors, the reprogramming efficiency was very low (<0.01% for newborn skin fibroblasts). This suggested that the intracellular modifications bound to the viral infection and/or the inflammatory microenvironment facilitated the iPSC induction by the transfected genes, whereas in non-infected cell populations only a small percentage of cells were permissive to the iPSC induction (elite versus the stochastic model for iPSC generation).

By now, iPSCs have been obtained in several laboratories by transfection with different associations of pluripotent genes [for a review, see (Li and Belmonte, 2017)]. From all studies, it appears that the transcription of *Oct4, Sox2*, and *Nanog* is crucial for the obtaining and maintenance of pluripotency. These factors form an autoregulatory loop by binding to each other’s promoters and activating their transcription. Other factors reported to play a significant role in the transcription factor network preserving cell pluripotency are L*in28,* KLF4, c-Myc, and Tcf3, a nuclear Wnt pathway component.

However, the overall picture of the network of involved genes is more complex. Epigenetic analyses revealed that regulatory networks underlying self-renewal and pluripotency are more intricate than previously realized. Analyzing hundreds of human iPSCs derived from different individuals, it was discovered a network of at least 13 genes highly correlated with each other and involved in the pluripotency acquisition, although the Oct4, Sox2, and Nanog complex remains the master regulator of pluripotency (Arthur et al., 2024).

## Genetic control of pluripotency *in vivo* and in cultures of early embryo-derived cells

*In vivo* and in cultures of early embryo cells, the pluripotency state is regulated by an interconnected network of pluripotency-related genes. Self-renewing naïve ESCs derived from the inner cell mass have different transcriptional signatures compared to post-implantation pluripotent cells. The epiblasts are self-renewing and pluripotent until gastrulation, when they undergo silencing of core transcription factor genes, activation of differentiation genes and progressive differentiation into the three germ layers and their derivatives.

The expression and the interaction of a core set of transcription factors is the necessary requirement to determine and stabilize cell pluripotency. Since the oocyte fertilization, during the development from zygote to gastrula epigenetic modifications of the chromatin organization, including methylation and nucleosome depletion, allow or do not allow the binding to their specific enhancers, promoters, and targets of transcription factors coded by pluripotency genes and other regulator proteins such as the Trithorax-group *proteins* (*TrxG*) and Polycomb (Pc) proteins. In several cases, this activates or inhibits classical signal transmission pathways and the response to extrinsic signals such as FGF, Wnt, and Activin/Nodal.

Despite the extensive investigation on the interactions among the transcription factors and their coding genes, the exact sequence order and the regulatory links in this network of pluripotent factors and genes is not fully elucidated yet. In this context, it should be noted that at different developmental stages, in several cases the same molecules exert different, and even opposite, functions (Furlan et al., 2023).

## Among pluripotency genes, *Oct4* is the more significant marker of cell stemness

The homeodomain transcription factor Oct4 (octamer-binding transcription factor 4 also named Oct 3/4 or Pouf1) is a major marker of cell stemness. Several isoforms of this factor exist. However, most studies focused on the more common Oct4A isoform. Oct4 is initially present as a maternal factor in the egg, both as protein and as mRNA, and is active in the embryo cells throughout the pre-implantation phase. Oct4 with Sox2 forms a heterodimer that binds to the Nanog promoter, determining its expression (Rodda et al., 2005). In turn, Oct4, Sox2, and Nanog form a complex promoting the transcription of genes coding for proteins playing a role in self-renewing and pluripotency maintenance (Boyer et al., 2005). Among these proteins, a major role is played by T-Cell Factor 3 (Tcf3), a downstream effector of the Wnt signaling pathway. Tcf3 regulates multiple lineage pathways controlling pluripotency and self-renewal of ESCs.

In human ESCs, the knockdown of Oct4 and Nanog genes by RNA interference promoted differentiation, thereby demonstrating a role for these factors in ESC self-renewal (Zaehres et al., 2005). Genome editing repressing *Oct4* caused a downregulation in the expression of genes associated with pre-implantation lineages, *Nanog* (epiblast), *Gata2* (trophectoderm), and *Gata4* (primitive endoderm) (Fogarty et al., 2017). Oct4 dosage is essential in determining the ESC fate. It was reported that quantitative expression of Oct4 defines differentiation, dedifferentiation, or self-renewal of ESCs. A less than two-fold increase in expression causes differentiation into primitive endoderm and mesoderm. On the contrary, repression of *Oct4* induces loss of pluripotency and dedifferentiation to trophectoderm (Niwa et al., 2000). In agreement with this finding, it was also observed that mouse embryos that are *Oct4* deficient or have low expression levels of Oct4 do not form an inner cell mass and differentiate into trophoectoderm (Nichols et al., 1998). By using an inducible CRISPR–Cas9 system and optimizing zygote microinjection, Fogarty et al. identified conditions that allowed to efficiently and precisely target *Oct4* in human zygotes. Live embryo imaging revealed that germ development was compromised and a deficient inner cell mass formation resulted in the subsequently embryo collapse (Fogarty et al., 2017). Transcriptomics analysis revealed that, in *Oct4*-null cells, gene expression was downregulated not only for extra-embryonic trophectoderm gene but also for regulators of the pluripotent epiblast, including *Nanog*. Tcf3 is critical for maintaining the appropriate levels of Oct4 and Nanog by repressing their expression. [(Boyer et al., 2005); see also following review sections].

Oct4, in addition to be expressed in totipotent ESCs and germ cells, may have a postnatal counterpart in the adult stem cells, recently described in various mammalian tissues. Oct-4 expression in putative stem cells purified from adult tissues has been considered a real marker of stemness (Zangrossi et al., 2007). Interestingly, *in vitro*, the transition of cells expressing Oct4 to a Transit Amplifying Cell (TAC) state is accompanied by the loss of Oct4 expression (*Muraglia, Cancedda, Mastrogiacomo manuscript in preparation*).

Oct4 not only maintains pluripotency in embryonic and adult stem cells but is also involved in the regulation of tumor cell proliferation and can be found in various cancers such as pancreatic, lung, liver and testicular germ cell tumors in adult germ cells (Saha et al., 2018). It is re-expressed in cancer stem tumor cell clusters observed in cancer relapses and chemotherapy resistance. Another effect this gene can have is to promote dysplastic growth in epithelial tissues when there is an ectopic expression of Oct4 within the epithelial cells (Hochedlinger et al., 2005).

## Sox2 acts synergistically with Oct4 to induce and maintain pluripotency

Sox2 also known as SRY-box 2 (sex determining region Y-box 2), is a member of the Sox transcription factor family crucial for maintaining self-renewal, and pluripotency of stem cells. Sox2 is induced by Oct4, and in turn Sox2 controls Oct4 expression in ESCs. The two factors act synergistically to activate Oct-Sox enhancers. Sox2 binds to DNA cooperatively with Oct4 at non-palindromic sequences to activate transcription of key pluripotency factors which regulate the expression of pluripotent genes, including *Nanog*, *Oct4* and *Sox2* itself (Masui et al., 2007). Interestingly, regulation of Oct4-Sox2 enhancers can occur without Sox2, possibly also due to the involvement of the most closely related Sox family members, Sox1 and Sox3 (Sarkar and Hochedlinger, 2013; Chambers and Tomlinson, 2009). Sox2 and Oct4 are each essential for mammalian development, since not only they control the transcription of genes crucial for development, but they also influence their own transcription through positive and negative feedback loops. Moreover, small variations in the Sox2 or Oct4 levels can promote the differentiation of ESCs (Rizzino, 2009).

Sox2 is required to regulate several transcription factors that affect *Oct4* expression and stabilizes ESCs in a pluripotent state by maintaining a correct level of Oct4 expression. In turn the forced expression of *Oct4* rescued the pluripotency of *Sox2*-null ES cells (Masui et al., 2007). Sox2 together with Oct4 and Nanog is part of a molecular complex that governs other genes controlling pluripotency. Epigenetic chromatin remodeling mechanisms, including DNA methylation and nucleosome depletion, facilitate the binding of these transcription factors to their enhancers. When Oct4 and Sox2 bind to the nucleosome-depleted regions, they promote transcription, whereas the subsequent progressive increase in DNA methylation leads to a persistent repression of Oct4 and Sox2 production and activity.

## Nanog is the third component of the master transcription factor complex controlling pluripotency

*Nanog* encodes an NK2-family homeobox transcription factor. It is the third component of the Oct4 and Sox2 complex controlling pluripotency gene expression. Sox2 and Oct4 drive the transcription of *Nanog*. Within the *Nanog* proximal promoter is present a sox-oct cis-regulatory element essential for *Nanog* pluripotent transcription. Using chromatin immunoprecipitation, Rodda et al. showed that Oct4 and Sox2 bind to the *Nanog* promoter in living mouse and human ESCs (Rodda et al., 2005). Furthermore, by RNA interference knockdown of Oct4 and Sox2 mRNA in ESCs, they provided also evidence for an interaction between Oct4, Sox2, and the *Nanog* promoter. Despite that Nanog and Oct4 are crucial in maintaining pluripotency in ES and EpiS cells, the specific interactions and their combined effects on cell pluripotency is still less understood. By investigating association of Nanog with other stem cell proteins, Roshan et al. evidenced distinct yet overlapping roles of Nanog and Oct4 in maintaining pluripotency in ESCs and EpiSCs (Roshan et al., 2024). The differential binding patterns and functional interactions between these factors underline the complexity of pluripotency regulation in different stem cell states.

Nanog protein also controls the expression of several genes essential in the pre-implantation development phase. *Nanog* expression progressively decreases during ESC differentiation, thus suggesting a role in the *in vivo* regulation of embryonic and fetal development. *Nanog*-null embryos die after implantation in accordance with the role of this gene and its coded transcription factor during pre-implantation development. Wang et al. described cell-line-specific requirements for Nanog, Oct4, and Sox2 in human ESCs (Wang et al., 2012). High levels of Oct4 specify mesendoderm in the presence of BMP4 whereas low levels of Oct4 induce embryonic ectoderm differentiation in the absence of BMP4. Nanog represses embryonic ectoderm differentiation and has little effect on other lineages. Thus, each factor controls specific cell fates.

In mouse ESCs, LIF-STAT3 signaling pathway is essential for their self-renewal. The *Nanog* forced expression allows the mouse ESC self-renewal also in the absence of LIF. *Nanog*-null ESCs can self-renew, although with a higher frequency of spontaneous differentiation. It is to note that, on the contrary, the forced expression of *Oct4* and/or *Sox2* cannot replace the need of LIF to sustain self-renewal of mouse ESCs.

The coordinated expression of pluripotency markers leading to the different tissues defines epiblast identity. Epiblasts, at variance with the embryo inner cell mass cells, present a cell-to-cell random variability in expression of different pluripotency markers. How the epiblast lineage specification initiates is still unclear. However, the process involves the activity of *Nanog*, together with genes such as *Gata6* and the FGF pathway. Coordination of pluripotency marker expression fails in *Nanog* and *Gata6* double KO embryos (Allègre et al., 2022).

It should also mentioned that Nanog as a transcription factor, is one of the most critical markers in Cancer Stem Cells (CSCs) and regulates multiple malignant phenotypes by modulating different signaling pathways such as AKT, STAT3 and P53 pathways (Vasefifar et al., 2022).

## The ancillary players Klf4 and Tcf3

Klf4 and Tcf3 are among the factors whose expression is induced by the Oct4, Sox2, Nanog complex. However, they exert a feedback control on the transcription of the genes coding for the complex proteins (Papatsenko et al., 2018).

Klf4 (Kruppel-like factor 4; Gut-enriched Krüppel-like factor or GKLF) is a member of the zinc finger KLF family of transcription factors. Klf4 is involved in the regulation in different cells of several apparently contradictory cell activities such as proliferation, apoptosis, and differentiation. However, there is a general consensus that Klf4 is highly expressed in non-dividing cells and its overexpression induces cell cycle arrest, in particular when the DNA is damaged (Ghaleb and Yang, 2017). Flow cytometric analyses indicated that the inducible expression of Klf4 caused a block in the G1/S phase transition of the cell cycle (Chen et al., 2001).

Loss of the transcription factor Tcf3 (a major effector of the Wnt signaling pathway) by RNA interference knockdown arrests ESC differentiation. In the pre-implantation embryo, Tcf3 expression is coregulated with Oct4 and Nanog in the blastocyst inner cell mass (Tam et al., 2008). Moreover many of the target genes of Oct4 and Nanog are also target genes of Tcf3 (Boyer et al., 2005).

## c-Myc isoforms

The Myc family proteins, originally identified because of their cancer-inducing capacity are transcription factors possessing a basic helix-loop-helix leucine zipper (bHLH-LZ). Myc forms heterodimers with its bHLH-LZ partner Max that binds to DNA sequences containing the E box CACGTG motif and either activates or represses transcription. In normal human cells, transcripts of the proto-oncogene *c-Myc* start at alternative promoters and encode for three protein isoforms named c-Myc-1 (67 kDa), c-Myc-2 (64 kDa), and c-Myc-S (55 kDa) (Hann et al., 1988; Spotts et al., 1997). The isoforms contain the same carboxy-terminal domain but differ in their N-terminal regions.

Regardless of the discovery for its involvement in cancer, c-Myc plays a major role in the control of stemness. C-Myc is instrumental in maintaining the pluripotency of ESCs by ensuring that these cells can differentiate into different cell lineage types. However, despite intensive studies, the mechanisms through which the different Myc isoforms act in diverse cellular processes remain poorly understood. Myc-2 is predominant in growing cells and shows oncogenic properties; this Myc isoform transactivates via the canonical EMS sequence and fails to transactivate the EFII enhancer element. On the contrary, Myc-1 has growth inhibitory properties; this Myc isoform is a potent and specific transactivator of the enhancer element EFII and is preferentially expressed when cells approach high-density growth arrest. Myc-1 is a strong inducer of apoptosis compared to Myc-2. The third isoform Myc-S is transiently expressed during rapid cell growth. For a comprehensive review on isoform-directed control of c-Myc functions see a recent review by Kubickova et al. (2023). In normal mammalian cells, Myc isoforms are produced in specific combinations or ratios characteristic of the specific cell status. Stoichiometric balance between Myc-1 and Myc-2 is important for regulation of cell proliferation.

How *Myc* expression is regulated to control pluripotency and early expression in embryo is still mostly unknown. Li-Bao et al. identified a DNA region that homes enhancers dedicated to *Myc* transcriptional regulation in stem cells of the pre-implantation and early post-implantation embryo and uncovered a modular mechanism for the regulation of Myc expression in different states of pluripotency. This region presents a cluster of elements exclusively dedicated to *Myc* regulation in pluripotent cells, with distinct enhancers that sequentially activate during naive to formative development.

When iPS cells are produced, the inclusion of *c-Myc* together with *Klf4* in the transfected genes might synergize with Thomson’s 4 factors (Oct4, Sox2, NANOG, Lin28) to reprogram the human somatic cells. Liao et al. showed that a combination of 6 transcription factors, Oct4, Nanog, Sox2, Lin28, C-Myc and Klf4, significantly increases the efficiency of generating iPS cells from human somatic cells (Liao et al., 2008). However, nature and properties of the transfected cells remain critical. Muse cells (Multi-lineage differentiating stress enduring cells) are a very rare sub-population of Mesenchymal Stem Cells (MSCs) self-renewing, and pluripotent (Minatoguchi et al., 2024). When human MSCs were transduced with *Oct4, Sox2, Klf4*, and *c-Myc*, iPSCs were derived from Muse cells but not from non-Muse cells (Wakao et al., 2011). Moreover, the correct balance of gene expression is also very important. A too high expression of c-Myc can have a toxic effect on the cell survival during the first week of culture.

## *Lin28*, a gene coding for a pluripotency epigenetic factor

Lin28 homolog A is a RNA-binding protein encoded by the *Lin28* gene. Lin28 acts as a pluripotency epigenetic factor reprogramming translation and promoting cancer progression. Lin28, originally identified through a mutant of the *Caenorhabditis elegans*, is a regulator of developmental timing in this nematode and controls stem cell self-renewal in other invertebrates. In mice, Lin28 protein is detectable in diverse tissues of the embryo through the period of organogenesis and it persists in some adult tissues (Campo) (Yang and Moss, 2003).

It plays an important role in the epigenetic control of cell proliferation. Lin28 blocks some microRNA maturation and also binds directly to a large number of cytoplasmic mRNAs thus reprogramming their translation. However, the exact molecular mechanisms remain unknown.

In humans, Lin28 is highly expressed during early embryogenesis, and its expression decreases during development and is lost in somatic cells after birth. Lin28 is highly expressed in ESCs (Richards et al., 2004) and enhances the efficiency of the formation of iPS cells from human somatic cells (Yu et al., 2007). Exogenous Lin28 is also required for the maintenance of self-renewal and pluripotency in presumptive porcine iPSCs (Chakritbudsabong et al., 2021).

Moreover, Lin28 expression is observed in several human cancers. Hence, Lin28 is considered an oncogene. In ES and iPS cells, the high expression of the oncogenic RNA-binding Lin28, and, at the same time the absence of its antagonist, the tumor-suppressor microRNA (miRNA) let-7, is crucial for maintaining pluripotency (Gunaratne, 2009). Interesting, it has been reported that in Muse cells, a non-tumorigenic population of adult pluripotent cells, Lin28 is not expressed and let-7 is expressed at higher levels than in ES and iPS cells. In these cells, let-7 maintains pluripotency through the inhibition of the PI3K-AKT pathway, leading to the synthesis of the pluripotency regulator KLF4 as well as its downstream gene products, OCT 3/4, SOX2, and NANOG (Li et al., 2024).

## Induction and control of pluripotency gene expression (signaling molecules, receptors, and mediators)

A variety of extrinsic signals and their receptors and mediators are known to be important in promoting and retaining pluripotency of stem cells in both mouse and human. However, it should be recalled that substantial differences exist in mouse and human about both negative and positive molecular control of pluripotency gene expression.

We will mention here only some of the more relevant involved molecules and signaling pathways. The pharmacological control of these signaling pathways could make possible the development of new approaches to preserve cell pluripotency.

### LIF: a key player cytokine

LIF (Leukemia inhibitory factor) is a member of the interleukin-6 cytokine family. LIF is the best characterized cytokine essential for self-renewal of mouse ES cells (Burdon et al., 1999; Chambers and Smith, 2004). It utilizes a receptor formed by the LIF receptor beta and gp130 which is also used by other cytokines. This heterodimer activates receptor-associated Janus kinases (JAKs), which phosphorylate tyrosine 705 of STAT3. Phosphorylated STAT3 enters the nucleus and binds to promoters of target genes to regulate their expression. Self-renewal of mouse ES cells is promoted by phosphorylated STAT3 also in the absence of LIF but, in this case, the presence of other serum components is necessary (Niwa et al., 1998; Matsuda et al., 1999).

The activation of these signal transduction pathways can have opposite effects in different cell types, such as stimulation or inhibition of cell proliferation and differentiation. Using an episomal super transfection of a mutant STAT3, Niwa et al. established that STAT3 plays a central role in the maintenance of the pluripotential stem cell phenotype. This contrasts with the involvement of STAT3 in the differentiation induction of somatic cells (Campo) (Niwa et al., 1998; Nicola and Babon, 2015). Moreover, Nicola and Babon showed that during ordinary mouse embryo development, LIF is important in maternal receptivity to blastocyst implantation, placental formation and in the development of the nervous system.

There is clear evidence that between mouse and human ESCs exist significant differences in response to cytokine signaling regarding *Oct4* regulation. In contrast to mouse ESCs, in human ESCs the LIF-STAT3 signaling pathway is active but not necessarily for activation of *Oct4* and their self-renewal (Daheron et al., 2004; Humphrey et al., 2004). In fact, in human ESCs transcriptional activation of *Oct4* and self-renewal appear more dependent on other cytokines, including FGF 2 (Chase and Firpo, 2007).

The activation of the LIF effector JAK-STAT3 is essential and sufficient to transfer LIF signals. However, the relationship between the activation of the LIF pathway and the Oct4, Sox2, Nanog complex is still only partially understood. Two LIF signaling pathways are connected to the complex via different transcription factors. In mouse ESCs, the transcription factor Klf4 is mainly activated by and preferentially activates *Sox2*, whereas Tbx3, whose activation is mainly controlled by the phosphatidylinositol-3-OH kinase-Akt and other kinase pathways, preferentially induces *Nanog* expression.

A different induction of Klf4 or Tbx3 is sufficient to maintain the expression of Oct4 and the cell pluripotency also in absence of LIF. Also to note that the overexpression of Nanog supports LIF-independent self-renewal of mouse ESCs without the need of Klf4 and Tbx3 activity. Therefore, Klf4 and Tbx3 play a role in the transfer of LIF signaling to the Oct4, Sox2, Nanog complex but are not directly involved with the maintenance of pluripotency because ESCs can keep pluripotency also in their absence (Niwa et al., 2009).

### Cytokines determining the early embryo axes are also crucial for specification of ESC pluripotency

**FIGURE 4:**
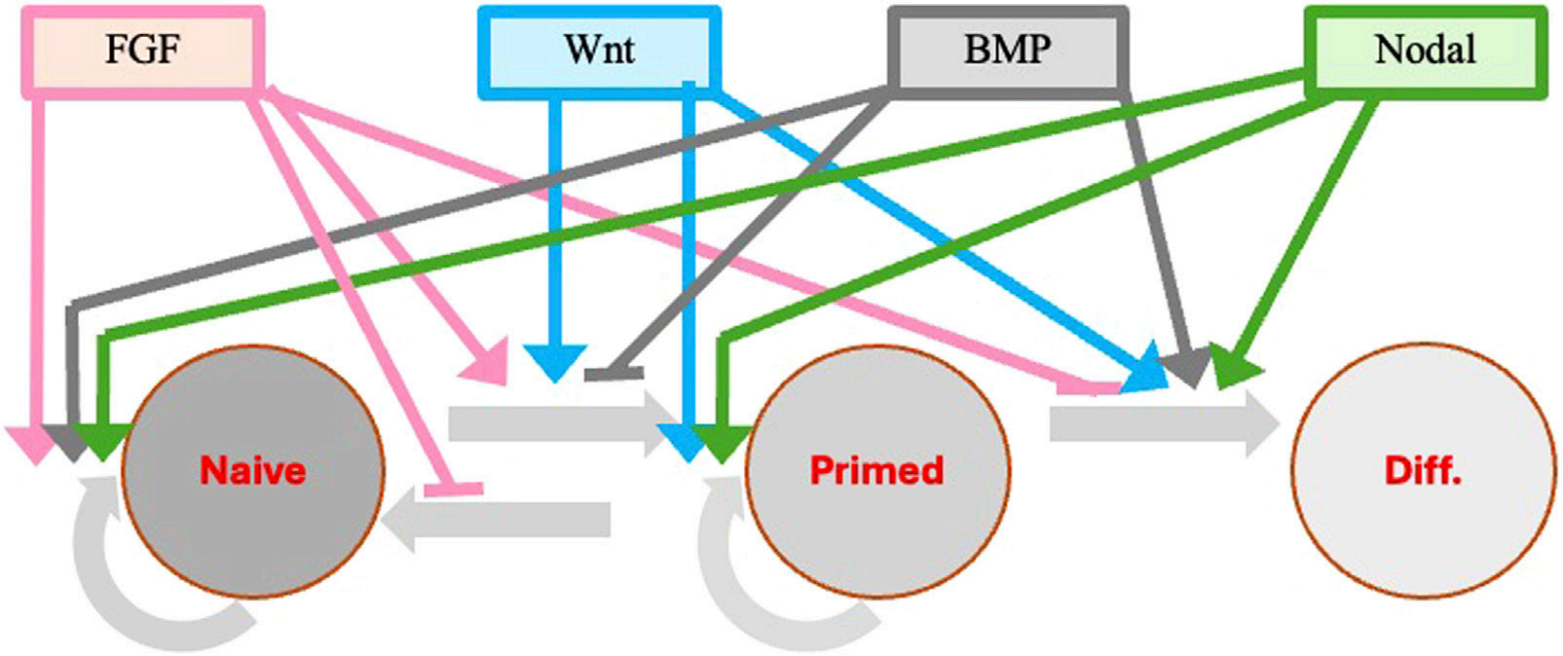
Modulation of pluripotency genes in response to extrinsic stimuli during the naïve to primed pluripotency transition. Extrinsic signals are important in promoting and retaining pluripotency of stem cells in both mouse and human. The same cytokines (FGF, Wnt, BMP, Nodal) that are involved in the designation of the embryo dorso-ventral and antero-posterior axes and in the positioning of the three germ layers at the time of gastrulation are also playing a major role in ESC self-renewal and progression through the different pluripotency stages. The transition from the primed ESCs and EpiSCs to progenitors specific to the different tissue lineages derived from each germ layer is also under the control of different additional cytokines and factors specific for each lineage (not shown). Colored continuous lines with rows refer to cytokine activation, whereas the same lines with small horizontal bars refer to inhibitory activity.

The main body plan of embryos is similar in all vertebrates and involves the designation of the dorsal-ventral and antero-posterior axes and the positioning of the three germ layers (ectoderm, mesoderm and endoderm) during gastrulation. This is mainly obtained through cell-cell interactions mediated by major embryonic signaling such as the FGF, Wnt, BMP, and Nodal pathways (Arnold and Robertson, 2009; Rossant and Tam, 2009; Molè et al., 2020). The same cytokines are also playing a major role in ESCs self-renewal and differentiation (Figure 4).

#### FGF

Fibroblast Growth Factor (FGF) is a family of 22 members sharing 30%–50% aminoacidic homology and characterized by a high affinity to heparin and heparan sulfate. The prototypes are acidic FGF (FGF 1) and basic FGF (FGF 2). FGF signaling is activated by a ligand-receptor interaction that results in the auto-phosphorylation of tyrosine residues in the intracellular region of four FGF receptors (FGFR 1–4). The signal is further transmitted through four distinct pathways: the Janus kinase/signal transducer and transcription activator (JAK-STAT) the phosphoinositide phospholipase C (PLCgamma), the phosphatidylinositol 3-kinase (PI3K), and the Erk pathways.

FGF/Erk signaling plays a major role in the control of pluripotency and lineage specification in different stem cell states, including the separation of pluripotent epiblasts and primitive endoderm in the blastocyst, the lineage priming of mouse ESCs, and the stabilization of mouse EpiSCs and human ESCs. Mouse and human ESCs are both derived from the pluripotent cells of inner cell mass but exhibit major differences regarding their FGF dependency. In the mouse ESCs, a more naïve pre-implantation state, the FGF/Erk signaling promotes the transition to a primed state. Autocrine FGF is the major stimulus activating Erk in mouse ES cells. Interference with FGF or Erk activity using chemical inhibitors or genetic ablations does not prevent propagation of undifferentiated ES cells. Instead, such manipulations restrict the ability of ES cells to commit to differentiation (Kunath et al., 2007). In human ESCs, an already primed post-implantation state, FGF/Erk signaling stabilizes the primed cells and prevents their reversion back to a naïve state (Brons et al., 2007; Nichols and Smith, 2009; Hanna et al., 2010). In human ESCs, FGF/Erk signaling maintains the pluripotent state and blocks neuronal, trophectoderm and primitive endoderm differentiation. In mouse EpiSCs, FGF/Erk signaling has a dual function: preventing neuronal differentiation and blocking reversion back to a pre-implantation ES cell-like state (Greber et al., 2010). Inhibition of FGF/Erk signaling release the reversion block. Klf4 transcript is involved in the block-release-processes. FGF/Erk inhibits the expression of endogenous Klf4 in EpiSCs. The EpiSCs to ESC-like state is released also by an overexpression of Klf4 (Guo et al., 2009). Therefore, human ESCs and EpiSCs require exogenous FGF for their derivation and maintenance, whereas mouse ESCs can be derived and maintained as such without exogenous FGF.

FGF also regulates Nanog expression both directly and indirectly via activin/Nodal induction in the feeder cells (Yu et al., 2011). Nanog protein can control the expression several genes crucial for the pre-implantation development phase. *Nanog* expression progressively decreases during ESC differentiation in agreement with its involvement in preventing differentiation to occurs.

Additional information on the FGF action in the initial phase of embryogenesis and in cultures of embryo derived ESCs can be found in two more extended and detailed reviews (Lanner and Rossant, 2010; Mossahebi-Mohammadi et al., 2020).

#### WNT

Wnt is a family of secreted proteins of 350–400 amino acids highly conserved within species acting as ligands in both a paracrine and autocrine modality. Before secretion, Wnts are glycosylated and palmitoleic acid is added to a conserved cysteine residue. Wnt signaling plays a major role in vertebrate embryo lineage specification in with sometimes opposite outcomes thus contributing to generate embryonic cell diversity (Sokol, 2011). Moreover, Wnt promotes self-renewal of pluripotent stem cells as well as differentiation (Nusse et al., 2008; Wend et al. 2010). This discrepancy could depend on the existence of the possible pre-implantation, primed, and intermediate different stages of ESCs (Sonavane and Willert, 2023).

Two of the three characterized Wnt signaling pathways involve the protein beta-catenin (canonical pathways). The Wnt/β-catenin pathway includes four elements: the extracellular ligand and the membrane, cytoplasmic, and nuclear elements. The Wnt ligands in mammals are coded by 19 genes and include Wnt3a, Wnt1, and Wnt5a. The main cell membrane receptor is composed of a Frizzled (a sevenfold transmembrane protein) forming a heterodimer with a low-density lipoprotein (LRP5/6). The cytoplasmic segment mainly includes beta-catenin. Its stability is controlled by a destruction complex (DC). Where the protein Axin acts as the scaffold, interacting with beta-catenin, the tumor suppressor APC, and two constitutively active serine-threonine kinases. In the absence of a Wnt signal, the destruction complex phosphorylates beta-catenin, targeting it for degradation by the proteasome whereas in the presence of the Wnt signal there is an accumulation of cytoplasmic beta-catenin. When the cytoplasmic beta-catenin concentration is high, the protein translocates to the nucleus where it engages DNA-bound T-cell factor/lymphoid enhancer factors (TCF transcription factors), thus promoting or inhibiting the transcription of target genes, such as matrix metallo-proteases (MMPs) and c-Myc (Nusse and Clevers, 2017).

Different studies provide conflicting evidence that, in human ESCs, Wnt/beta-catenin promotes either self-renewal or differentiation. However, inhibition of Wnt/beta-catenin signaling had no negative effect on ESC maintenance over multiple passages, whereas its activation led to a loss of self-renewal and an induction of mesoderm lineage genes (Davidson et al., 2012). In agreement with the above, in the same cells, Oct4 repressed beta-catenin signaling during self-renewal and knockdown of *Oct4* activated beta-catenin (Faunes et al., 2013). Moreover, it was also shown that human ESCs with elevated beta-catenin signaling expressed higher levels of differentiation markers (Davidson et al., 2012). On the contrary, in human ESCs in a naïve stage, Wnt/beta-catenin signaling promoted the self-renewal of the naïve ESCs. In conditions that inhibit Wnt/beta-catenin signaling, naïve human ESCs remained undifferentiated, although they showed a more primed-like protein expression profile (Xu et al., 2016). The mechanisms by which Wnt signaling acts as a regulator of transitions between pluripotent states are still partially known, although in all cases they are leading to the inactivation of TCF-mediated repression (Sonavane and Willert, 2023; Sokol, 2011). TCF proteins repress gene targets in the absence of the Wnt signal, but upon association with beta-catenin are converted into activators of transcription.

The culture of mouse ESCs in the presence of LIF could replace the need for beta-catenin in ESC self-renewal. Wnt also upregulates the signal transducer STAT3, a downstream effector of LIF suggesting a link, between Wnt and LIF signaling (Hao et al., 2006; Ogawa et al., 2006). In fact, the requirement for LIF is reduced in the presence of Wnt pathway inhibitors (Wray et al., 2011).

Additional information on the Wnt action can be found in more extended and detailed reviews (Sokol, 2011; Holzem et al., 2024; Liu et al., 2022).

#### BMP

Bone morphogenetic proteins (BMPs), originally identified as osteoinductive components in extracts derived from bone, are now known to be involved in a wide array of processes during formation and maintenance of various organs. So far, more than a dozen BMPs have been identified in vertebrates. These BMPs have highly conserved structures common to the members of the TGF-beta family. Based on structural homology, the BMPs can be further classified into several subgroups, including BMP 2,4 group, BMP 5,6,7 (OP-1), BMP 8 group, BMP 9,10 group, and BMP 12,13,14 (GDF 5,6,7) group. Like other members of the TGF-beta family, BMPs act as ligand for type I and type II serine-threonine kinase receptors and their intracellular downstream effectors include Smad proteins. Unlike other TGF-beta family members, BMPs are capable of binding to type I receptors in the absence of type II receptors. However, the binding affinities is much higher when both type I and type II receptors are present (Rosenzweig et al., 1995). BMP-activated type I receptors phosphorylate Smad effectors (Smad1, Smad5, and Smad8) at their carboxy-terminal. These phosphorylated Smads form complexes with Smad4 (common partner to all Smads) and move into the nucleus where they associate with various transcriptional co-activators or co-repressors to activate or repress the expression of target genes.

BMPs play an important role in several processes during embryo development, morphogenesis, and adult homeostasis. They regulate cell lineage commitment, differentiation, proliferation and apoptosis of different types of cells. In the early embryo BMP is a key morphogen with a role in dorsal-ventral patterning. The mechanisms by which BMP control patterning in the embryo are still the object of studies. It was very recently reported that in a cell model for patterning of the human embryo, pluripotent cells respond more strongly to variations in BMP duration of signaling than in its level. However, both level and duration of signaling activity control cell fate choices by changing the time integral (Teague et al., 2024).

In ESC cultures, BMP signaling sustains self-renewal of mouse naïve ESCs. BMP-4 is crucial, together with LIF, to maintain mouse ESCs in their undifferentiated pluripotent state. BMP signaling has negative effects on mouse ESC neural differentiation (Zhang et al., 2010). On the contrary, BMP induces differentiation of human-primed ESCs.

It was proposed that mouse ESC neural differentiation occurs in two stages: first from ESCs to EpiSCs and then from EpiSCs to neural precursor cells. Apparently, in the first stage, BMP4 is inhibitory for the ESCs to EpiSCs conversion, and suppresses EpiSC neural commitment by promoting non-neural lineage differentiation in the second stage. Endogenous Wnt signals both are BMP targets required and sufficient for mesoderm induction and induce loss of pluripotency both in human ESCs and in their murine EpiSC counterparts. On the contrary, Wnt inhibition stabilizes a stem cell in the pre-gastrula state with the ability to contribute to blastocyst chimeras (Kurek et al., 2015).

The treatment with an inhibitor of the BMP pathway maintained the pluripotency and homogeneity of porcine ESCs for an extended period in the absence of feeder cells by stimulating the secretion of chemokines and suppressing differentiation, (Choi et al., 2024).

The SMAD1 and SMAD5 transducers of BMP signals, in association with KLF4 and KLF5, recognize enhancer regions in naive mouse ESCs. KLF4 physically interacts with SMAD1 and suppress its activity. In agreement, *Smad1/5*double-knockout mouse ESCs remained in the naive state, indicating that the BMP-SMAD pathway is not an absolute requirement for it. However, double Smad1 and Smad5 knockout mouse ESCs were still pluripotent, but they exhibited higher levels of DNA methylation than their wild-type counterparts and had a higher propensity to differentiate (Gomes Fernandes et al., 2016).

In contrast, the MEK5-ERK5 pathway mediated the BMP-4 induced self-renewal of mouse ESCs by inducing *Klf2*, a critical factor for the pluripotency state (Morikawa et al., 2016).

A more detailed recent review for role of BMP biological mechanism is in (Kalal and Mohapatra, 2025).

#### Nodal/activin

Nodal is a TGF-beta superfamily member, conserved across evolution, which plays key roles in early embryo development. In mammals, Nodal is translated as a precursor with a large N-terminal domain which is cleaved by proteases for activity. Active Nodal proteins act as regulators of pluripotency in the peri-implantation period and control axis formation and gastrulation (Robertson, 2014). Nodal has a role in primitive streak formation and specification of mesoderm and endoderm. Nodal signaling is critical for both the maintenance of pluripotency and the differentiation of human pluripotent cells. Pluripotency maintenance *in vitro* is dependent upon exogenous Nodal, but an endogenous Nodal signaling pathway is part of an autocrine pathway for the regulation of human pluripotent cell self-renewal.

Nodal *in vivo* forms a heterodimer with GDF3, another TGF-beta superfamily member. The mature active Nodal interacts with type 1 and 2 serine/threonine kinase receptors. The type 1 receptor phosphorylates the type 2 receptor, which in turn phosphorylates SMAD2 and 3, acting as co-activators of tissue specific transcription factors (Hayes et al., 2021). *Nanog* is a target of SMAD2 signaling in human pluripotent cells, as in mouse EpiSCs, and is responsible for repression of the neural differentiation pathway. Heterogeneity exists in human ESC cultures, and only a small subpopulation of cells is capable of self-renewal. This subpopulation not only expresses the highest levels of transcripts for *Nodal*, *GDF3,* nodal receptors, and other Nodal signaling elements but also expresses high levels of pluripotency associated transcription factors and display properties of the formative state.

A signaling pathway like the one of Nodal is used by the other TGF-beta superfamily member Activin. Because it is difficult to obtain an active recombinant Nodal protein, Activin or TGF-beta1 are often used as medium supplement to replace Nodal and in pluripotent cell cultures. Although Activin and Nodal signaling are often considered interchangeably, there are elements that function only in the Nodal pathway. The Nodal specific elements include the proteins Left y1 and 2, which antagonize only Nodal by interacting with Nodal or its coreceptor Tdgf1. Lefty and Nodal are part of a negative feedback loop in which Nodal induces the expression of its own inhibitor. Significantly, inhibition of Nodal signaling in the human preimplantation embryo, but not in the mouse, led to a loss of *Nanog* expression.

In knockout mouse embryos, *Nodal* deletion mutants arrest at the egg cylinder stage and die post implantation, before gastrulation. The epiblast itself loses pluripotency and goes into a precocious neural specification (Robertson, 2014). In mouse ESCs, FGF signaling and Nodal together with Smad2 and 3 are required for the transition from naïve to formative pluripotency state. After mouse ES cells have left the naïve state, they become competent for germline and somatic differentiation. *Nodal* loss affects the cell ability to give rise to mesoderm or endoderm cells. Similarly to the *in vivo*, pluripotency of these cultured cells is compromised and cells by default undergo a neural lineage differentiation (Mulas et al., 2017).

In summary, in the mouse Nodal is required for the transition from the naïve to the primed state and for the maintenance of primed pluripotency. A recent, complete review on Nodal can be found in (Hayes et al., 2021).

#### Epigenetic modifications and additional factors and cytokines involved in the induction and maintenance of pluripotency

Epigenetic modifications are changes in gene expression occurring without alteration of the DNA sequence. These modifications are primarily of two types: DNA methylation and histone modifications leading to a different chromatin organization. These modifications affect reading and expression of underlaying genes thus influencing cellular processes and development. The changes in chromatin accessibility and 3D chromatin organization can determine regulatory switches of gene transcription. Proteins codified by these genes include specific transcription factors, soluble modulators, such as growth factors and cytokines, and their receptors.

Preimplantation embryo development occurs in four stages: fertilization, cell cleavage, morula and blastocyst formation. Each stage is characterized by its own epigenetic profile and epigenetic factors that play crucial roles in determining the transition from each embryo stage to the subsequent stage. Maternal cytoplasmic components providing the signals for the initial cell divisions before transcription of the embryo’s own genes are unevenly distributed in the oocyte cytoplasm. These components include RNAs, proteins, and other molecules essential for fertilization, and early cell cycle progression. After fertilization, at the time of the initial cell cleavage, the association of nuclei in principle identical with different maternal cytoplasmic components determine the occurrence of different epigenetic modifications and gene expression of different genes by the different embryo cells. The consequent activation of autocrine and paracrine cell crosstalk triggers and supports embryo development. Epigenetic modifications also mark future events, particularly the lineage specification decisions at the time of gastrulation.

Studies of mammalian development have advanced our understanding of the epigenetic, and cellular processes that regulate early embryogenesis, stimulating new ideas about epigenetic reprogramming, cell fate control, and the potential mechanisms underpinning developmental plasticity in human embryos. These new findings are reported and discussed in two recent reviews (Xu et al., 2021; Wilkinson et al., 2023). A detailed description of all extrinsic and intracellular modulators controlling pluripotent gene expression and ESCs differentiation cannot be covered by this type of review. In the following paragraphs, we will briefly recall only some modifications and molecules that have been the objects of some more recent publications.

Epigenetic modifications and changes in response to extrinsic stimuli concur in the modulation of pluripotency genes to confer robustness during the naïve to primed pluripotency transition. Furlan et al. have recently proposed the notion of Molecular Versatility to regroup mechanisms by which molecules are repurposed to exert different, sometimes opposite, functions in close stem cell configuration (Furlan et al., 2023).

DNA methylation and histone reorganization are crucial in the activation or repression of specific genes controlling pluripotency in the early stages of embryo development. DNA methylation is closely associated with reprogramming, functional remodeling, and differentiation of pluripotent stem cells. Developmental plasticity of naive ESCs and EpiSCs is linked to genome hypomethylation. While LIF–Stat3 signaling induces genomic hypomethylation, genome methylation is dynamically controlled through modulation of alpha-ketoglutarate availability or Stat3 activation in mitochondria. Alpha-ketoglutarate links metabolism to the epigenome by reducing the expression of some target genes. Genetic inactivation of these genes results in genomic hypomethylation even in the absence of active LIF-Stat3. *Stat3* ^−/−^ ESCs show decreased alpha-ketoglutarate production from glutamine, leading to increased target gene expression and DNA methylation (Betto et al., 2021).

ESC pluripotency is regulated by transcription factors in concert with chromatin regulators. The multifunctional histone chaperone SET acts as a potent regulator of pluripotency in early differentiation. Through alternative promoters, SET shows a shift from the predominant SET-alpha isoform present in ESCs to the SET-beta isoform in differentiated cells (Edupuganti et al., 2017). The ESC and EpiSC propensity to preferentially differentiate toward one germ layer over the other germ layers depends on many complex factors. Using immuno-precipitation coupled with protein quantitation by LC-MS/MS, it was possible to uncover factors and complexes, including P53 and beta-catenin, by which SET regulates lineage specification. Knockdown for P53 in SET-knockout mouse ESCs partially rescued lineage marker misregulation during differentiation. Paradoxically, SET-knockout ESCs show a decreased expression of several Wnt target genes despite reduced levels of active beta-catenin. Overall, the discovery of a role for both P53 and beta-catenin in SET-regulated early differentiation suggested a SET function at the beta-catenin-Wnt regulatory axis (Harikumar et al., 2020).

The Estrogen Related Receptor Beta (ESRRB), one of the main proteins expressed as result of the epigenetic modifications, guides pluripotent cells through the initial phases of differentiation. In fact, the removal of its coding gene allows pluripotent stem cells to differentiate out of control. During embryonic development, naive pluripotent cells transit to a formative state. The formative epiblast cells form a polarized epithelium, exhibit distinct transcriptional and epigenetic profiles, and acquire competence to differentiate into all somatic and germline lineages. Although we still have limited knowledge of how the transition from the naïve to the formative state is molecularly controlled, it has been reported that ESRRB was both required and sufficient to activate formative genes in murine ESCs. Genetic inactivation of *Esrrb* leaded to illegitimate expression of mesendoderm and extra-embryonic markers, impaired formative expression, and resulted in failure to self-organize in 3D (Carbognin et al., 2023). Fluorescent *Nanog* and *Esrrb* reporters showed an Esrrb downregulation only in Nanog^low^ ESCs whereas other *Esrrb* reporters showed that Esrrb^negative^ ESCs cannot effectively self-renew (Festuccia et al., 2018).

The transition from the primed ESCs and EpiSCs to progenitors specific to the different tissue lineages derived from each germ layer is under the control of different cytokines and factors specific for each lineage. In recent years, the acquired knowledge has been utilized to develop protocols leading to the generation of differentiated cells starting from ESCs. Given the technical and ethical difficulties to derive cells from human blastocysts and gastrulas, in most cases, human differentiated cells have been obtained starting from iPSCs. Probably the most well established and reproducible protocols are the ones developed to obtain human neurons. An example of the molecular interactions occurring in these types of processes is the protocol for differentiating IPSCs into neurons called “Dual SMAD Inhibitors” (Chambers et al., 2009). Intracellular SMAD signaling proteins must be inhibited to ensure neuroectodermal patterning of iPSCs, and to do this, two separate molecules are used: an inhibitor of the TGF-beta pathway (SB431542) and an inhibitor of the BMP pathway (LDN-193189). The inhibition of these two signaling pathways is critical in neuronal induction since TGF-beta binding to its receptor leads to phosphorylation and activation of SMAD2 and SMAD3 proteins that mediate meso- and endodermal induction. Similarly, when BMP binds to its receptor, it leads to the activation of SMAD1, SMAD5 and SMAD8, which in turn promote mesodermal or ectodermal non-neural differentiation.

## Conclusion

All cells derived by the initial segmentation of the zygote have identical DNA genetic information. However, because of the association of nuclei with different regions of the maternal cytoplasm the embryo cells start to introduce different epigenetic modifications in their DNA. As results the early embryo cells express different genes and release different signaling molecules acting via autocrine and paracrine mode. This way the initial axis symmetries are generated and while some cells self-renew without big changes in their properties, other cells begin to follow different differentiation pathways. The transition from the self-renewing to the differentiation committed cells occurs through at least three phases: naïve stem cells (long-term pluripotent stem), primed stem cells (short-term pluripotent stem) and an intermediate phase (formative).

A network of a relatively small number of pluripotent genes acting directly or indirectly via specific effectors in different combinations and at different development stages can have completely opposite effects in controlling pluripotent stem cell self-renewal and pluripotent stem cell differentiation. A same cytokine or transcription factor can play a crucial role in maintaining self-renewing naïve ESCs and in promoting primed ESC differentiation toward different cell lineages. Mouse ESCs derived from the blastocyst inner cell mass present properties characteristic of naïve pluripotent stem cells, whereas ESCs derived from early embryo epiblasts (also named EpiSCs) are still pluripotent, i.e., they can generate cells of the three germ layers but have lost several of the naïve cell properties and have acquired properties of primed stem cells. At variance with mouse ESCs, human ESCs derived from the blastocyst inner cell mass already present properties of primed pluripotent stem cells, although, in certain culture conditions, they can revert to a self-renewal naïve state. Therefore, the same transcription factors can have completely opposite effects also in mouse and human ESCs.

## Author’s note

Given that in many cited articles the authors performed experiments with both mouse and human cells and that often it was not immediately obvious the cell species, we arbitrary decided to use through all text the regular style for both species. On the contrary, we respected the convention to write genes in italic style and proteins in regular style.
